# *Mycobacterium tuberculosis* Cell Wall Fragments Released upon Bacterial Contact with the Human Lung Mucosa Alter the Neutrophil Response to Infection

**DOI:** 10.3389/fimmu.2017.00307

**Published:** 2017-03-20

**Authors:** Julia M. Scordo, Jesús Arcos, Holden V. Kelley, Lauren Diangelo, Smitha J. Sasindran, Ellie Youngmin, Mark D. Wewers, Shu-Hua Wang, Joan-Miquel Balada-Llasat, Jordi B. Torrelles

**Affiliations:** ^1^Department of Microbial Infection and Immunity, Center for Microbial Interface Biology, College of Medicine, The Ohio State University, Columbus, OH, USA; ^2^Department of Internal Medicine, Pulmonary, Critical Care & Sleep Medicine Division, College of Medicine, The Ohio State University, Columbus, OH, USA; ^3^Department of Internal Medicine, Infectious Disease Division, College of Medicine, The Ohio State University, Columbus, OH, USA; ^4^Department of Pathology, College of Medicine, The Ohio State University, Columbus, OH, USA

**Keywords:** tuberculosis, neutrophil, lung mucosa, innate immunity, cell wall

## Abstract

In 2016, the World Health Organization reported that one person dies of tuberculosis (TB) every 21 s. A host environment that *Mycobacterium tuberculosis* (*M.tb*) finds during its route of infection is the lung mucosa bathing the alveolar space located in the deepest regions of the lungs. We published that human lung mucosa, or alveolar lining fluid (ALF), contains an array of hydrolytic enzymes that can significantly alter the *M.tb* surface during infection by cleaving off parts of its cell wall. This interaction results in two different outcomes: modifications on the *M.tb* cell wall surface and release of *M.tb* cell wall fragments into the environment. Typically, one of the first host immune cells at the site of *M.tb* infection is the neutrophil. Neutrophils can mount an extracellular and intracellular innate immune response to *M.tb* during infection. We hypothesized that exposure of neutrophils to ALF-induced *M.tb* released cell wall fragments would prime neutrophils to control *M.tb* infection better. Our results show that ALF fragments activate neutrophils leading to an increased production of inflammatory cytokines and oxidative radicals. However, neutrophil exposure to these fragments reduces production of chemoattractants (i.e., interleukin-8), and degranulation, with the subsequent reduction of myeloperoxidase release, and does not induce cytotoxicity. Unexpectedly, these ALF fragment-derived modulations in neutrophil activity do not further, either positively or negatively, contribute to the intracellular control of *M.tb* growth during infection. However, secreted products from neutrophils primed with ALF fragments are capable of regulating the activity of resting macrophages. These results indicate that ALF-induced *M.tb* fragments could further contribute to the control of *M.tb* growth and local killing by resident neutrophils by switching on the total oxidative response and limiting migration of neutrophils to the infection site.

## Introduction

Despite the strides made in the tuberculosis (TB) research field over the years, TB remains a major public health concern (1, 2). *Mycobacterium tuberculosis* (*M.tb*) is transmitted primarily *via* the aerosol route and is delivered into the distal lung space. In order to understand the host cell-*M.tb* interaction, it is imperative to study the environment, which *M.tb* encounters following inhalation.

Our lab has shown that the lung microenvironment plays a role in altering the interaction between *M.tb* and host cells in the alveolar space (3, 4). Specifically, we showed that the human lung mucosa, or alveolar lining fluid (ALF), contains hydrolases that significantly alter the *M.tb* cell wall surface (4). We have demonstrated that cell surface alterations on *M.tb* after exposure to human ALF enhance the killing capacity of phagocytes (3, 4). In addition to altering the *M.tb* bacterial surface, *M.tb* exposure to ALF releases *M.tb* cell wall fragments into the lung milieu (4). It is thus plausible that these released *M.tb* cell wall fragments may contact host cells in the lung space both prior to and during *M.tb* infection.

In blood, neutrophils are 60% of the total host leukocyte population (5), and they are abundant in sputum and bronchoalveolar lavage fluid (BALF) from active TB patients (6). Neutrophils are one of the first innate effector cells to arrive at the site of infection, and published reports show beneficial (i.e., bacterial growth control), as well as detrimental (i.e., excessive oxidation and tissue damage) roles for neutrophils during *M.tb* infection (7). Additionally, reports are inconclusive as to the killing capacity of neutrophils following *M.tb* phagocytosis (7) or show a limited role during infection *in vivo* (8).

Contact with *M.tb* results in neutrophil phenotypic changes including increased reactive oxygen species (ROS) production, secretion of chemokines and cytokines, and acquisition of migratory abilities to secondary lymphoid organs (8–10). *M.tb* can survive the oxidative burst in neutrophils leading to bacterial growth, tissue destruction and bacterial dissemination (11–13). Interestingly, our lab recently showed that when *M.tb* is exposed to the human ALF and encounters neutrophils, these phagocytes can better control infection through enhanced intracellular killing mechanisms and dampening of both extracellular killing mechanisms and inflammatory responses (3).

Here, we extended these studies and examined if released *M.tb* cell wall fragments during *M.tb* exposure to human ALF play a collaborative role in regulating the neutrophil’s activation and subsequent control of infection. We found that ALF-induced *M.tb* cell wall released fragments (ALF fragments), in the absence of *M.tb*, activated neutrophils, increased their respiratory burst, tumor necrosis factor (TNF), and interleukin (IL)-10 production, yet decreased chemoattractant release (i.e., IL-8) and did not induce their degranulation. Importantly, ALF fragment-primed neutrophils did not further alter their capacity to recognize and kill *M.tb*; however, this control of *M.tb* seems toward an increase of the oxidative burst in detriment of the phagosome–lysosome killing mechanism. Collectively, these results support a role for the human lung mucosa in locally positively influencing the host control of *M.tb* during infection.

## Materials and Methods

### Human Subjects

This study was carried out in accordance with the recommendations of US Code of Federal and Local Regulations (University IRB protocol numbers 2008H0135 and 2008H0119), and Good Clinical Practice as approved by the National Institutes of Health/National Institute of Allergies and Infectious Diseases/Division of Microbiology and Infectious Diseases (NIH/NIAID/DMID protocol number 12-0086) with written informed consent from all subjects. All subjects gave written informed consent in accordance with the Declaration of Helsinki. The protocol was approved by the OSU IRB for Human Subjects.

### Reagents and Antibodies

All reagents were from Sigma-Aldrich (St. Louis, MO, USA) except the following: human TNF, IL-6, IL-8, IL-1β, IL-12p40, myeloperoxidase (MPO), and IL-10 enzyme-linked immunosorbent assay (ELISA) kits (R&D Systems); phycoerythrin (PE)-mouse anti-human CD63 antibody (Ab), PE-mouse antihuman CD11b-Ab, PerCP/Cy5.5-mouse antihuman CD11c-Ab, FITC-mouse antihuman CD62L-Ab, allophycocyanin (APC)-mouse antihuman CD206 [mannose receptor (MR)]-Ab, FITC-mouse antihuman CD80-Ab, PE-mouse antihuman CD86-Ab, PerCP/Cy5.5-mouse antihuman HLA-DR-Ab, PE-mouse immunoglobulin (Ig) G_1_ ĸ isotype control, PerCP/Cy5.5-mouse IgG2a ĸ isotype control, FITC-mouse IgG1 ĸ isotype control, and APC-mouse IgG1 ĸ isotype control-Ab (BD Biosciences); APC-mouse antihuman CD35 and peridinin chlorophyll protein–cyanine 5.5 (PerCP/Cy5.5)-mouse antihuman CD66b antibodies, and their respective isotype controls (Biolegend); 5 (and 6)-carboxy-2′,7′-dichlorofluorescein diacetate (DCF) (Invitrogen); human gene expression primers for IL-8 and TNF (Invitrogen); PE Annexin V Apoptosis Detection Kit (BD Biosciences); and CytoTox 96^®^ Non-Radioactive Cytotoxicity Assay kit (Promega).

### *M.tb* Growth

GFP-*M.tb* Erdman (obtained from Dr. Marcus Horwitz, UCLA) was grown as we previously described (4). Briefly, *M.tb* was grown for 9–14 days on 7H11 plates supplemented with oleic acid-albumin-catalase-dextrose (OADC) enrichment at 37°C, 5% CO_2_.

### Human ALF Isolation

Alveolar lining fluid was obtained from human BALF as previously described (3, 4, 14). Briefly, BALF (a total of BALFs from 15 individual human donors were used in this study) was obtained by BAL in 80 mL sterile 0.9% NaCl (following an approved IRB protocol at OSU and NIH/NIAID/DMID protocol), concentrated 20-fold by using a 10,000 molecular mass cut off (MMCO) membrane Centricon Plus (Millipore) device at 4°C to achieve the ALF volume present within the lungs. This removed surfactant lipids (free or in micelles) leaving the functional hydrolases in ALF (>10 kDa MMCO). Lipid removal from ALF was confirmed by mass spectrometry of the fatty acid methyl ester derivatives (data not shown). Protein content [performed by the BCA method following manufacturer’s instructions (BioRad, Hercules, CA, USA)] and phospholipid (14) content were also determined in crude surfactant and ALF. ALF (defined in this study as BALF > 10 kDa fraction) was frozen at −80°C until use.

### Isolation and Preparation of Human Neutrophils and Day 5 Peripheral Blood Mononuclear Cells (PBMCs) Containing Monocyte-Derived Macrophages (MDMs)

Human neutrophils were obtained from healthy human donors as we have previously described (3). Briefly, heparinized blood was centrifuged at 450 × *g* for 1 h at room temperature using density centrifugation to separate PBMCs. PBMCs were removed, and the resulting pellet, containing mostly neutrophils and red blood cells, was further separated using dextran gradient fractionation at 4°C for 20–60 min. The resulting supernatant, containing mainly neutrophils, was removed, pooled in 50 mL conical tubes, and centrifuged at 750 × *g* for 10 min at 4°C. Remaining red blood cells were lysed by addition of sterile water for 30 s, isotonicity was restored by the addition of an equal volume of sterile 1.8% NaCl, and cells were pelleted by centrifugation at 150 × *g* for 5 min at 4°C. This process was repeated until purified neutrophils were isolated, yielding a purity of 97–99% as observed by microscopy following Giemsa stain. Neutrophils were then suspended in Hank’s buffered salt solution (HBSS), counted, and kept on ice until immediate use. Day 5 PBMCs containing MDMs were obtained and prepared as previously described (4). For all experiments, “*n*” value represents number of different human donors used to obtain human neutrophils or PBMCs.

### Preparation of Exposed *M.tb* and Generation of Fragments

Single cell suspension of *M.tb* (1 × 10^8^) was achieved as described (3), and further exposed to either human ALF at its physiological concentration within the human lung (4) or to sterile 0.9% NaCl for 12 h at 37°C, 5% CO_2_ as we previously described (3, 4, 14). After incubation, bacilli were gently centrifuged and the supernatant containing released fragments filter-sterilized for immediate use. Bacilli were washed and suspended in HBSS immediately prior to infection. Infections were performed using *M.tb* counted in a Petroff Hausser chamber. *M.tb* cell wall fragments were used in experiments at a multiplicity of exposure (MOE) as indicated. MOE is defined as the ratio of fragments being released from a given number of *M.tb* bacilli relative to the number of human neutrophils being used, i.e., MOE 20:1 is defined as 2.5 × 10^5^ neutrophils exposed to fragments derived from 5.0 × 10^6^ *M.tb* bacilli. For the majority of experiments, exposed *M.tb* and/or their released fragments were freshly made. In some occasions, exposed *M.tb* fragments were aliquoted and stored at −80°C until use. We did not observe any variation in our results as a consequence of freezing the *M.tb* released fragments and generated fragments are reproducible independent of the ALF used (14). Each set of experiments was performed with fragments derived from using at least two different human ALFs, with the number of ALFs used in each experiment listed in the figure legend. ALF-*M.tb* fragments are never pooled.

### Neutrophil Exposure to *M.tb* Fragments

Human neutrophils were exposed to *M.tb*-derived fragments at an MOE of 20:1, unless stated otherwise. MOE 20:1 is defined as 2.5 × 10^5^ neutrophils exposed to fragments derived from 5.0 × 10^6^ *M.tb* bacilli. In some experiments, neutrophils were simultaneously infected with either ALF- or 0.9% NaCl-exposed *M.tb* (i.e., exposed *M.tb* in the presence of their respective fragments) at the multiplicity of infection (MOI) (number of exposed *M.tb* to number of neutrophils) as indicated. For these infection studies, the MOI and MOE are the same. All exposure and/or infections were performed at 37°C, 5% CO_2_ for the times indicated, depending on experimental design. For all exposed *M.tb* fragment exposures, neutrophils were exposed to ALF control or 0.9% NaCl in parallel to account for background levels.

In order to simplify the presentation of the data as we used multiple ALFs for each “*n*” value (where “*n*” represents number of human primary cell donors), we opted to subtract all the proper backgrounds and controls from our two groups studied (0.9% NaCl fragments vs. ALF fragments). Proper controls included (i) resting neutrophils; (ii) neutrophils exposed to 0.9% NaCl alone (as a control for 0.9% NaCl fragments), and (iii) neutrophils exposed to human ALF alone (as a control for ALF fragments). In all the parameters studied, controls had low values (i.e., equal to that observed with resting neutrophils).

### Neutrophil Respiratory Burst (ROS)

Neutrophil production of ROS was determined by two methods: (1) fluorescence by conversion of non-fluorescent 2′,7′-dichlorodihydrofluorescein diacetate (H2DCFDA) to the highly fluorescent 2′,7′-dichlorofluorescein (DCF) as previously described (3); and (2) luminol-dependent chemiluminescence. Briefly, exposed and/or infected neutrophils were incubated with 20 μM DCF or 500 μM luminol, and respiratory burst was assessed every 12 min for 2 h by a Spectramax GEMINI-EM (Molecular Devices, Sunnyvale, CA, USA) plate reader set to 37°C.

### Neutrophil Degranulation and Release of MPO

Degranulation by human neutrophils was assessed by flow cytometry as we previously described (3). Briefly, neutrophil-fragment exposure (for 2 h) and/or infection were terminated, and cells were stained with antibodies specific for cell-surface degranulation markers (PE-mouse antihuman CD63-Ab, PerCP/Cy5.5-mouse antihuman CD66b-Ab, and APC-mouse antihuman/NHP CD35-Ab) for 30 min on ice in dark conditions. Neutrophil surface expression of CD63, CD66b, and CD35, markers of primary, secondary, and tertiary granules, respectively, was assessed by counting ≥10,000 events. All appropriate isotype controls were included (PE-mouse IgG1 ĸ isotype control-Ab, PerCP/Cy5.5-mouse IgM ĸ isotype control-Ab, and APC-mouse IgG1 ĸ isotype control-Ab). Samples were read on a BD FACS CANTO II flow cytometer (BD Biosciences, San Jose, CA, USA), and data were analyzed using BD FACS Diva software. MPO release in neutrophil cell supernatants was detected by ELISA per kit instructions (R&D Systems, Minneapolis, MN, USA).

### Neutrophil Surface Receptor Expression and Activation

Human neutrophil surface expression of activation markers and phagocytic receptors was assessed by flow cytometry (15). Briefly, fragment-primed neutrophils (for 2 h) were stained with antibodies specific for cell activation and surface receptors (PE-mouse antihuman CD11b-Ab, PerCP/Cy5.5-mouse antihuman CD11c-Ab, and FITC-mouse antihuman CD62L-Ab) for 30 min on ice in dark conditions. Neutrophil surface expression was assessed by counting ≥10,000 events. All appropriate isotype controls were included (PE-mouse IgG1 ĸ isotype control-Ab, PerCP/Cy5.5-mouse IgG2a ĸ isotype control-Ab and FITC-mouse IgG1 ĸ isotype control-Ab). Samples were read on a BD FACS CANTO II flow cytometer (BD Biosciences, San Jose, CA, USA), and data were analyzed using FlowJo™ Version 9.7.6 Software (FlowJo, LLC, Ashland, OR, USA).

### Association of Exposed *M.tb* with Human Neutrophils

Association (combined cell surface binding and bacterial uptake) of exposed *M.tb* with neutrophils was performed at an MOI of 10:1 for 10 or 30 min at 37°C in the presence or absence of their respective fragments. Association was determined by counting the number of *M.tb* bacilli associated with ≥500 consecutive neutrophils per coverslip performed in replicate using phase-contrast and fluorescence microscopy, as previously described (3).

### *M.tb*-Infected Neutrophil Total and Intracellular Killing

Neutrophil-*M.tb* killing experiments were performed as previously described (3). Briefly, neutrophils were plated and infected for 30 min with ALF- or 0.9% NaCl-exposed *M.tb* (MOI 1:1) in the presence of their respective fragments. After the infection period, infected neutrophils were washed and lysed with cold 1% Triton X-100 in PBS for 5 min. Cell lysates were then serially diluted 10-fold in 7H9 + OADC medium, plated, and cultured on 7H11 plates for 21 days at 37°C for colony forming units (CFU) determination. For determination of intracellular killing, *M.tb*-infected neutrophil monolayers were washed and further incubated with 50 μg/mL of gentamicin for 30 min at 37°C and 5% CO_2_ to kill cell-associated extracellular *M.tb* (3) that did not get phagocytized by neutrophils. After gentamicin incubation, neutrophils were lysed as mentioned above and lysates plated for CFU determination. For TNF preactivation studies, neutrophils were preincubated with 10 ng/mL of human TNF for 30 min prior to infection with exposed *M.tb* (MOI 1:1) in the presence or absence of their respective fragment for an additional 30 min (3).

### Neutrophil mRNA Expression

Neutrophils infected with human ALF- or 0.9% NaCl-treated *M.tb* in the presence of their respective fragments were lysed after 6 h, neutrophil total RNA was isolated using TRIzol reagent, and cDNA was transcribed using Superscript III Reverse Transcriptase. Gene expression was determined by real-time quantitative PCR using a TaqMan gene expression system (Invitrogen) with gene-specific primers to quantify human TNF, IL-6, and IL-8 mRNA expression using a BioRad CFX96 Real-Time System (BioRad, Hercules, CA). All samples were analyzed in duplicate. Negative controls included in qRT-PCR reactions included sample with no reverse transcriptase and no cDNA template. Samples were normalized to β-actin, and fold change was calculated relative to uninfected neutrophil expression values.

### *M.tb*-Infected Neutrophil Phagosome Maturation

Neutrophils, on coverslips, were infected with human ALF- or 0.9% NaCl-treated *M.tb* at an MOI of 10:1 for 30 min at 37°C and 5% CO_2_ in the presence or absence of their respective exposed *M.tb* fragments. Following infection, coverslips were washed with PBS, fixed with 2% paraformaldehyde, permeabilized, blocked, and stained with the integral membrane protein marker CD63 (0.5 μg/mL) or IgG_1_ isotype control. Phagosome maturation events were imaged by confocal microscopy and quantified by counting the number of GFP-*M.tb*^+^ phagosomes that colocalized with CD63, representing the number of phagosome–lysosome (P–L) fusion events. P–L fusion events were represented as fold change of ALF-*M.tb*-infected neutrophils in the presence or absence of ALF fragments vs. 0.9% NaCl-exposed *M.tb*-infected neutrophils in the presence or absence of 0.9% NaCl fragments. For all experiments >150 consecutive phagosomes per coverslip, in replicate, per test group were counted.

### Determination of Neutrophil Programmed Cell Death

Neutrophil apoptosis was determined by flow cytometry using a BD Annexin V Apoptosis Detection Kit (BD Biosciences). Briefly, neutrophil-fragment exposure and/or infection were terminated and cells were incubated with PE-Annexin V Ab (binds cell surface-exposed phosphatidylcholine) and 7-amino-actinomycin D live/dead stain for 15 min in dark conditions. Cells were fixed with 4% paraformaldehyde and ≥10,000 events read on a BD FACS CANTO II or a BD LSR II (BD Biosciences, San Jose, CA, USA), and data were analyzed using BD FACS Diva software for quantification of early and late apoptosis.

### Neutrophil Cytotoxicity

Neutrophil cell death/necrosis was assessed by measuring the amount of lactate dehydrogenase present in cell supernatants using a CytoTox 96^®^ Non-Radioactive Cytotoxicity Assay kit (Promega, Madison, WI, USA).

### Neutrophil Released Immune Modulators of Macrophage Activation

Day 5 PBMCs were prepared as described (4). Following preparation, 5 × 10^6^ PBMCs were added to 5 mL polypropylene tubes in low volume RPMI + 2% autologous serum. PBMCs were primed with 0.22 μM-filtered ALF fragment or 0.9% NaCl fragment exposed neutrophil supernatants for 24 h at 37°C, 5% CO_2_. Priming was terminated by centrifugation and all supernatants were collected and stored at −20°C. Day 5 PBMCs were stained for markers of macrophage activation (APC-mouse antihuman CD206-Ab, FITC-mouse antihuman CD80-Ab, PE-mouse antihuman CD86-Ab, and PerCP/Cy5.5-mouse antihuman HLA-DR-Ab) on ice for 30 min in dark conditions, fixed with 2% paraformaldehyde and ≥10,000 events read on a BD FACS CANTO II or a BD LSR II (BD Biosciences, San Jose, CA, USA). All appropriate isotype controls were included (APC-mouse IgG1 ĸ isotype control-Ab, FITC-mouse IgG1 ĸ isotype control-Ab, PE-mouse IgG1 ĸ isotype control-Ab, and PerCP/Cy5.5-mouse IgG_2a_ ĸ isotype control-Ab). Data were analyzed using FlowJo Version 9.7.6 Software for mean fluorescence intensity of activation marker surface expression. The MDM population was gated based on FSC and SSC parameters as well as cell-specific markers. The following controls were included in all experiments: (i) positive control-primed MDMs (10 ng/mL LPS); (ii) resting macrophages in RPMI; (iii) resting macrophages exposed to supernatants from neutrophils exposed to 0.9% NaCl; and (iv) resting macrophages exposed to supernatants from neutrophils exposed to human ALF. Control values were low and have been subtracted from figures for clarity.

### Phagocyte Cytokine Production

Cytokine release in human neutrophil and Day 5 PBMC containing MDMs supernatants was determined using ELISA per kit instructions (R&D Systems, Minneapolis, MN, USA). For supernatants collected from neutrophil-PBMC cross talk studies, neutrophil only production of cytokines was subtracted from all experimental conditions.

### Statistics

Statistical analyses were performed using GraphPad 4.0/5.0 Prism software. To determine the statistical significance between the means for two experimental groups, an unpaired, two-tailed Student’s *t*-test was performed. For comparisons of the means between more than two experimental groups with a single independent variable, one-way ANOVA with post-Tukey analyses were performed. Differences were considered statistically significant at a *p < 0.05; **p < 0.005; ***p < 0.0005 or at a ^§^*p* < 0.05; ^§§^*p* < 0.005; ^§§§^*p* < 0.0005.

## Results

### Neutrophil Activation Modulated by the Action of *M.tb* Fragments Released Upon Exposure to the Human Lung Mucosa

**Figure 1:**
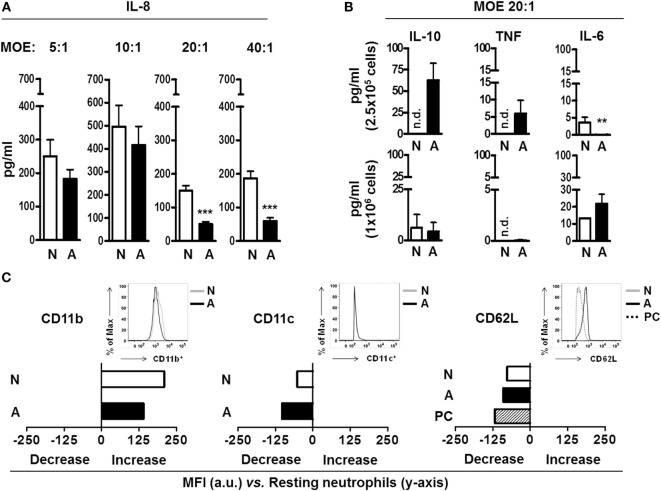
**Neutrophil immune response and activation status after being exposed to alveolar lining fluid (ALF) fragments**. **(A)** Neutrophils were exposed to fragments for 18 h at different multiplicity of exposure (MOE), and production of interleukin (IL)-8 was measured by enzyme-linked immunosorbent assay (ELISA) (*n* = 3 with six to eight ALFs). **(B)** Neutrophils were exposed to fragments for 18 h at MOE of 20:1, and IL-10, tumor necrosis factor (TNF), and IL-6 production was measured by ELISA. Top is *n* = 3–4 with three ALFs, shown per 2.5 × 10^5^ cells; bottom is *n* = 1 with three ALFs, shown per 1.0 × 10^6^ cells. **(C)** Mean fluorescence intensity (MFI) of the **s**urface expression of phagocytic markers CD11b (*n* = 4 with three ALFs), CD11c (*n* = 3 with two ALFs), and the activation marker CD62L (*n* = 3 with three ALFs) on neutrophils exposed to fragments for 2 h at MOE 20:1; shown in bar graphs are data of change in MFI (increase or decrease) from one representative experiment vs. naïve neutrophils (*y*-axis); insets showing histograms from one representative experiment. Isotype control values have been subtracted. For all graphs, Student’s *t*-test comparing neutrophils exposed to ALF fragments vs. 0.9% NaCl fragments; **p < 0.005; ***p < 0.0005. N: 0.9% NaCl fragments; A: ALF fragments; PC: positive control [*N*-formyl-Met–Leu–Phe (1 μg/mL) stimulated neutrophils and phorbol 12-myristate 13-acetate (PMA; 10 ng/mL)-stimulated neutrophils]; n.d.: not detected; a.u.: arbitrary units. Values for controls (0.9% NaCl alone as a control for 0.9% NaCl fragments and human ALF alone as a control for ALF fragments) were low and subtracted from all graphs.

Neutrophils are the most numerous cell type found in BAL from active TB patients (6). Neutrophil chemotaxis is driven in part by the immune mediator IL-8, produced by several cell types not limited to neutrophils, macrophages and alveolar epithelial cells (16). We sought to determine if fragments released from the *M.tb* surface after ALF exposure could alter neutrophil IL-8 production. As shown in Figure 1A, we observed a decrease in release of IL-8, which was independent of the length of exposure (2–18 h, data not shown) but dose-dependent with the most significant reduction at an MOE of 20:1 (fragments released from *M.tb* bacilli: neutrophils ratio, shown the 18 h time point, for other time points data not shown). While we observed a trend in decreased IL-8 release by neutrophils exposed to ALF-*M.tb* fragments at all MOEs tested, the significance observed at an MOE of 20:1 and 40:1 prompted us to utilize the MOE 20:1 (the minimum MOE providing us significant differences between the tested groups) for all our subsequent studies. In contrast to these findings, ALF fragments failed to induce significant differences in TNF, IL-6, or IL-10 compared to 0.9% NaCl fragments (control) over background levels of protein produced by naïve neutrophils. Our observations of low protein production by fragments over resting values were independent of the number of neutrophils used for the exposure (Figure 1B, 2.5 × 10^5^ neutrophils or 1 × 10^6^ neutrophils).

We next assessed if fragments could alter the surface receptor expression and activation status of neutrophils prior to their encounter with *M.tb*. We chose to focus on neutrophil expression of CD11b (or complement receptor 3, CR3), CD11c (or CR4), and CD62L (L-Selectin) as these receptors are important for neutrophil phagocytosis, migration, and activation (17). Our results (Figure 1C) show that both ALF- and 0.9% NaCl fragments exposure at MOE 20:1 altered the basal neutrophil surface expression of CD11b and CD11c. However, when ALF- and 0.9% NaCl fragments were compared for their effects on neutrophils, differences between them were minimal. Conversely, exposure to ALF fragments activated neutrophils as indicated by the lower levels of CD62L on their surface (18) was similar to our positive control (exposure to phorbol 12-myristate 13-acetate or PMA). This decrease was not observed for 0.9% NaCl fragments. These data indicate ALF fragments may modulate resting neutrophil function through altering their surface receptor expression and activation status.

**Figure 2:**
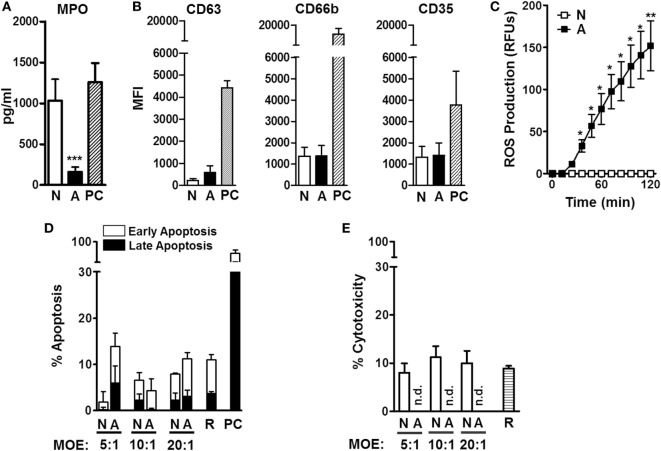
**Neutrophil degranulation, oxidative response, and cell death after being exposed to alveolar lining fluid (ALF) fragments**. **(A)** Neutrophils were exposed to fragments at multiplicity of exposure (MOE) 20:1 for 18 h, and release of myeloperoxidase (MPO) in medium was measured from supernatants by enzyme-linked immunosorbent assay (*n* = 3 with three ALFs). **(B)** Neutrophils were exposed to fragments at MOE 20:1 for 2 h, and release of primary (CD63), secondary (CD66b), and tertiary (CD35) granules was measured by flow cytometry (*n* = 4 with three ALFs). **(C)** Neutrophils were exposed to fragments at MOE 20:1, and oxidative response was measured *via* reactive oxygen species production (*n* = 4 with three ALFs). **(D)** Neutrophil early and late apoptosis 2 h following fragment exposure at various MOEs (up to 40:1, see Figure S1A in Supplementary Material) (*n* = 2 with two ALFs). **(E)** Neutrophil cytotoxicity by lactate dehydrogenase release 18 h following fragment exposure at various MOEs (up to 20:1). Percent cytotoxicity data are shown vs. lysis positive control, *n* = 3 with three ALFs. For all graphs, Student’s *t*-test comparing neutrophils exposed to ALF fragments vs. 0.9% NaCl fragments; *p < 0.05; ***p < 0.0005. N: 0.9% NaCl fragments; A: ALF fragments; R: resting neutrophils; PC: positive control [phorbol myristate acetate (PMA; 10 ng/mL) or *N*-formyl-Met–Leu–Phe (1 μg/mL)-stimulated neutrophils]; n.d.: not detected. Values for controls (0.9% NaCl alone as a control for 0.9% NaCl fragments and human ALF alone as a control for ALF fragments) were low and subtracted from all graphs.

Neutrophils are equipped with defense mechanisms to combat pathogens, such as toxic granules and ROS (17). We next sought to examine neutrophil extracellular degranulation through release of MPO, contained in primary granules, and surface expression of CD63, CD66b, and CD35, markers of primary, secondary, and tertiary granules, respectively. Our results indicate that ALF fragments significantly reduced neutrophil release of MPO (Figure 2A). Moreover, we did not observe differences in neutrophil degranulation after exposure to ALF fragments or control 0.9% NaCl fragments, as measured by cell surface expression of specific primary, secondary, and tertiary granule markers by flow cytometry (Figure 2B). Finally, we measured the neutrophil respiratory burst by the conversion of the non-fluorescent dye H_2_DCFDA to the fluorescent dye DCF as a measure of both intracellular/intraphagosomal and extracellular release of ROS (total ROS). Interestingly, when neutrophils were exposed to ALF fragments, we found significantly increased levels of ROS (Figure 2C). We also determined that ALF fragments did not induce apoptosis and/or cytotoxicity (Figures 2D,E; Figures S1A,B in Supplementary Material). Collectively, *M.tb* fragments released upon exposure to human ALF could balance the neutrophil’s activation through a decrease of chemokine production, and extracellular degranulation, while simultaneously inducing the respiratory burst.

### *M.tb* Fragments Impact the Capacity of Neutrophils to Handle the Infection

**Figure 3:**
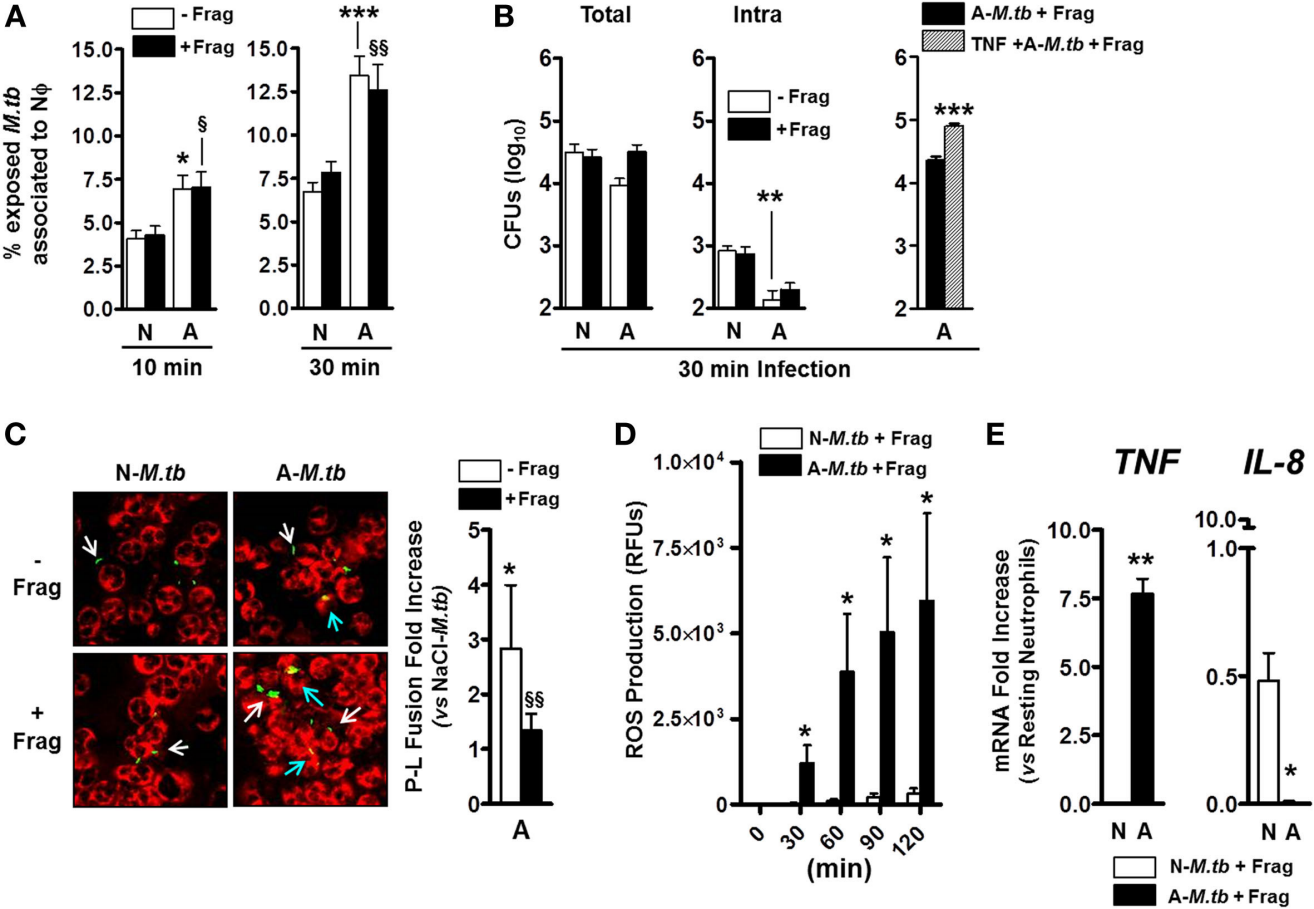
**Effects of alveolar lining fluid (ALF) fragments on neutrophils during *Mycobacterium tuberculosis* (*M.tb*) infection**. Neutrophils were infected with ALF-exposed or 0.9% NaCl-exposed *M.tb* in the presence or absence of their respective fragments. **(A)** % of *M.tb* associated to neutrophils on coverslips after infection for 10 min (left) or 30 min (right) at multiplicity of infection (MOI) 10:1. Association (binding and uptake) was determined by fluorescence microscopy (*n* = 4 with four ALFs for 10 min; *n* = 5 with five ALFs for 30 min). **(B)** Exposed *M.tb* growth following 30 min neutrophil infection at MOI 1:1. Total (left) and intracellular (middle) *M.tb* growth in the presence or absence of their respective fragments. Total growth (right) in tumor necrosis factor (TNF) preactivated neutrophils (30 min preactivation with 10 ng/mL human TNF) present an initial worse control of ALF-*M.tb* infection (0.56log_10_ increase in total growth) when compared to resting neutrophils (*n* = 2 with two ALFs). **(C)** Colocalization of phagosomes containing ALF- or 0.9% NaCl-exposed GPF-*M.tb* (green) with lysosomes (CD63 positive compartments, red) in the presence or absence of fragments by confocal microscopy (where colocalization or P–L fusion is yellow). Fold increase of P–L fusion for ALF-exposed *M.tb* with respect to 0.9% NaCl-exposed *M.tb* in the presence (black bar) or absence (white bar) of ALF fragments following 30 min neutrophil infection at MOI 10:1 (*n* = 4 with four ALFs). White arrows indicate GFP-*M.tb* not fused with CD63, and blue arrows indicate GFP-*M.tb* fused with CD63 (P–L fusion event). **(D)** Reactive oxygen species generation by neutrophils infected at MOI 10:1 with 0.9% NaCl- or ALF-exposed *M.tb* in the presence of their respective fragments (*n* = 2 with two ALFs). **(E)** TNF and interleukin (IL)-8 gene expressions by neutrophils infected for 30 min at MOI 10:1 with 0.9% NaCl- or ALF-exposed *M.tb* in the presence of their respective fragments. Gene expression determined by qRT-PCR at 6 h post-*M.tb* infection and represented as relative mRNA expression vs. resting neutrophils (*n* = 2 with two ALFs). For panels **(A–C)**, one-way ANOVA post-Tukey comparing between groups: neutrophils infected with ALF-*M.tb* or 0.9% NaCl-*M.tb* in the presence or absence of their corresponding fragments; A: ALF-exposed *M.tb* and N: 0.9% NaCl-exposed *M.tb*-infected neutrophils (white bars, *p < 0.05; **p < 0.005; ***p < 0.0005) in the presence of their respective fragments (black bars, **^§^***p* < 0.05; **^§§^***p* < 0.005); *ns*: non-significant for panels **(D,E)**, Student’s *t*-test comparing neutrophils infected with ALF-*M.tb* vs. 0.9% NaCl-*M.tb* in the presence of their corresponding fragments; *p < 0.05; **p < 0.005. A: ALF-exposed *M.tb* + fragments; N: 0.9% NaCl-exposed *M.tb* + fragments.

Our lab has previously shown that *M.tb* exposure to human ALF modifies the *M.tb* cell wall (4), and this results in enhanced neutrophil killing of *M.tb* (3). Thus, we aimed to extend these studies and determine whether altered neutrophil activation status induced by contact with ALF fragments (Figures 1 and 2) impacted the outcome of *M.tb* infection. In this context, it is plausible that locally both released fragments and ALF-modified *M.tb* may contact neutrophils during infection altering *M.tb* association with neutrophils. Our results show that independent of the presence of ALF fragments, ALF-exposed *M.tb* associated at higher levels than our control 0.9% NaCl-exposed *M.tb* in the presence of 0.9% NaCl fragments (Figure 3A).

Furthermore, ALF-exposed *M.tb* could be controlled better (0.52log_10_) by neutrophils (Figure 3B, comparing white bars between groups in the total and intracellular growth); however, neutrophils priming with ALF fragments failed to increase their intracellular killing capacity (Figure 3B, comparing white vs. black bars within groups; middle graph). In other words, although we observed enhanced intracellular bacterial growth control in the presence of ALF fragments, there were no differences in comparison to what we observed with ALF-exposed *M.tb* alone (Figure 3B, middle graph). Unexpectedly exposure of neutrophils to ALF fragments followed by infection, while not significant, negatively impacted the total killing capacity of the neutrophil (Figure 3B, comparing white vs. black bars; left graph). Moreover, this decreased capacity of controlling ALF-*M.tb* total growth in the presence of ALF fragments was further exacerbated when neutrophils were preactivated with TNF (Figure 3B, right graph).

To further assess why the presence of fragments failed to further enhance the intracellular killing capacity of the neutrophil, we assessed the neutrophil’s killing mechanisms that could be altered by exposure to ALF-*M.tb* fragments. Following phagocytosis, neutrophil granules fuse and deliver antibacterial mediators to phagosomes promoting bacterial killing (19). In the context of *M.tb* infection, published reports on the antimycobacterial function of neutrophils are widely inconclusive (7). We previously showed that modifications on the *M.tb* cell wall due to the exposure to human ALF increases the neutrophil capacity to control *M.tb* intracellular growth *via* altered trafficking leading to increased phagosome maturation (3). Furthermore, we observe a P–L fusion fold increase in the presence of ALF fragments (Figure 3C, see black bar showing P–L fusion fold increase of ALF-*M.tb* plus fragments vs. NaCl-*M.tb* plus fragments). When directly comparing the effects of adding the fragments on the capacity of neutrophils to limit the growth of ALF-*M.tb*, we observed a decrease in P–L fusion, but this was not significant (Figure 3C, white vs. black bar) leading to loss of the neutrophil’s capacity to better control intracellular *M.tb* growth (Figure 3B). Importantly, all neutrophil phenotypes induced by the presence of ALF fragments alone (i.e., induction of neutrophil ROS and TNF, lack of neutrophil degranulation, apoptosis, and necrosis, and decrease of IL-8) were maintained during *M.tb* infection (Figures 3D,E; Figures S1C,D in Supplementary Material and data not shown).

### Crosstalk between Neutrophils Exposed to ALF-*M.tb* Fragments and Other Host Cells

**Figure 4:**
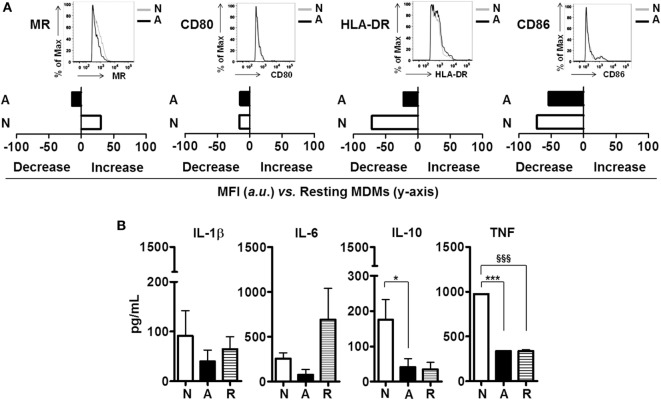
**Effects of alveolar lining fluid (ALF) fragments on neutrophils crosstalk with host cells**. Resting neutrophils were primed with ALF- or 0.9% NaCl fragments at MOE 20:1 for 18 h. Secreted products of resting (in Hank’s buffered salt solution) and primed neutrophils were collected, 0.22 μm filter-sterilized and incubated with resting day 5 peripheral blood mononuclear cells (PBMCs) containing monocyte-derived macrophages (MDMs) for 24 h. **(A)** Data from a representative flow cytometry experiment of *n* = 2–4 (using two ALFs) showing that exposure of resting MDMs to ALF fragment-primed neutrophil secretions altered the macrophage surface expression of the mannose receptor (MR), and co-stimulatory molecules (CD80/CD86); data shown in bar graphs are the change in mean fluorescence intensity (increase or decrease) of markers on the surface of MDMs vs. naïve MDMs (shown on *y*-axis); insets showing histograms from one representative experiment. Isotype control values have been subtracted. **(B)** Day 5 PBMCs containing MDMs cytokine responses to ALF- or 0.9% NaCl fragment-primed neutrophil secretions detected by enzyme-linked immunosorbent assay, *n* = 2–4 with two ALFs. For panel **(B)**, one-way ANOVA post-Tukey comparing macrophage responses to secretions from neutrophils primed with ALF fragments, 0.9% NaCl fragments and resting neutrophil secretions; *p < 0.05; ***p < 0.0005; ^§§§^*p* < 0.0005. N: resting macrophages exposed to secreted products from 0.9% NaCl fragment-primed neutrophils; A: resting macrophages exposed to secreted products from ALF fragment-primed neutrophils; R: resting macrophages exposed to secreted products from resting neutrophils. Values for controls (MDMs incubated with supernatant derived from neutrophils exposed to 0.9% NaCl alone and MDMs incubated with supernatant derived from neutrophils exposed to human ALF alone) were low and subtracted from all graphs.

We hypothesized that soluble immune mediators secreted by neutrophils primed with ALF-*M.tb* fragments could further modulate the responses of resting macrophages. Our results indicate that secreted products of ALF fragment-primed neutrophils were able to downregulate the macrophage surface expression of the MR, an important receptor involved in *M.tb* survival (20). However, these secreted products had limited effect on the macrophage activation status (Figure 4A, i.e., no significant effects on CD80/CD86/HLA-DR surface expression when compared to secreted products from neutrophils primed to 0.9% NaCl fragments). Importantly, secreted products of ALF fragment-primed neutrophils were also able to modulate the cytokine response of resting macrophages (Figure 4B). Specifically, a decrease of IL-6 secretion was observed when compared to the response induced by secreted products derived from resting neutrophils (R, resting cell control). Contrary to what was observed for secreted products of 0.9% NaCl fragment-primed neutrophils, secreted products by ALF fragment-primed neutrophils did not induce the secretion of IL-1β, IL-10, and TNF in resting macrophages.

## Discussion

The human lung mucosa (ALF) plays an important role in maintaining the homeostasis of the alveolar space (21); however, the functionality and complexity of ALF as a whole in determining the establishment of a lung infection is understudied. This is particularly important in the case of *M.tb* infection in which the alveolar space, which is bathed by ALF, is thought to be the environment where the first contact among *M.tb* and host cells occurs. We have shown that ALF contains a series of host hydrolases capable of modifying the *M.tb* cell envelope with two outcomes, alteration of the *M.tb* cell wall surface and release of cell wall fragments into the milieu (4). The *M.tb* cell wall alterations caused by these ALF hydrolases redefines the interaction of *M.tb* with phagocytes, allowing the latter to control the infection better (3, 4) and thus defining a new innate role for ALF components. In this study, we extended our findings examining the role of the *M.tb* cell wall fragments released upon exposure to ALF (ALF fragments) in dictating the neutrophil immune response, as well as the capacity of neutrophils exposed to these fragments to modulate the immune response of resting macrophages.

The importance of the neutrophil in the host innate response, and more specifically in *M.tb* infection, is well established. In this context, a healthy lung contains a very limited number of neutrophils (22); however, an inflamed lung, such as the one during active TB disease, contains a large number of neutrophils that can drive extensive tissue damage (23). Our previous studies showed that neutrophils could better control the total growth of ALF-exposed *M.tb* with enhanced intracellular killing mechanisms and dampened extracellular inflammatory responses (3). These studies, however, did not discern the role of ALF fragments on *M.tb*-neutrophil interactions. In the present study, our results indicate that ALF fragments modulate neutrophils by limiting their IL-8 (CXCL-8) production, an important mediator involved in neutrophil recruitment to the infection site (24). We observed this decrease in ALF fragment exposed neutrophil IL-8 production at all MOEs tested, although the magnitude of IL-8 by all groups tested was highest at lower MOEs tested. This could be explained by the solubility of fragments at these concentrations. The reduction in IL-8 production driven by ALF fragments is also maintained during *M.tb* infection and may be critical to limit excessive neutrophil migration. Additionally, ALF fragments induced the oxidative response and modulated phagocyte activation, but did not induce degranulation, apoptosis and/or cytotoxicity. Although there was a slight increase in CD63 expression following neutrophil exposure to ALF fragments, the difference was not significant and may have been a result of transient fluctuations in surface expression at the early time point chosen for these studies. In support of this, the low concentration of MPO observed in cell culture supernatants of neutrophils exposed to ALF fragments for 18 h is due to accumulation in the medium of extracellularly released MPO *via* degranulation after neutrophil activation (25). We show that ALF fragments have the ability to alter the receptor surface expression in resting neutrophils, altering the basal expression of CD11b/CD11c. The lower levels of CD11b expression on the neutrophil surface observed by ALF fragment vs. 0.9% NaCl fragments exposure may be explained by the decreased neutrophil release of MPO following exposure to ALF fragments. Independent of its catalytic activity, MPO has inflammatory cytokine activity that acts in a positive feedback mechanism to increase neutrophil integrin expression (such as CD11b/CD18), degranulation and MPO production (26). MPO-dependent integrin expression may have important implications in cell-to-cell interactions in the alveolar space (27). Finally, ALF fragments do not significantly alter neutrophil cytokine release (TNF, IL-6, and IL-10) in comparison to cytokines induced by neutrophil exposure to 0.9% NaCl fragments. Additionally, cytokine production above the levels produced by naïve neutrophils were low, independent of the number of neutrophils exposed to fragments.

We show here that human neutrophils exposed to ALF-*M.tb* fragments (and to a lesser extent 0.9% NaCl fragments) are capable of IL-10 release. While human neutrophil IL-10 production has been shown by a few groups to be inhibited due to inactivity of the IL-10 gene locus, others have shown human neutrophil IL-10 production in response to fungal pathogens (28) and through direct-cell contact (29). Differences in the findings shown here may be due to the stimuli used to activate human neutrophils. In this regard, ALF-*M.tb* fragments are a heterogeneous mixture of *M.tb* cell wall molecules that may be capable of neutrophil activation through interaction with several surface receptors (PRRs).

Unexpectedly, we observed that ALF fragments triggered a robust respiratory burst both in the case of resting and ALF-*M.tb*-infected neutrophils. Increased ALF fragment induced neutrophil total ROS production (intraphagosomal and/or extracellular) may be explained by our observations of low levels of MPO. In support of this, studies show MPO-deficient neutrophils have increased oxygen intake leading to enhanced superoxide production and phagocytosis. Furthermore, oxidative killing observed in neutrophils lacking MPO suggests multiple antibacterial mechanisms (30). Additional support for augmented neutrophil activation in response to ALF fragments is shown by the surface decrease of CD62L, a cell adhesion molecule that is shed from the surface of activated neutrophils (31). It is therefore plausible that selective changes in neutrophil activation following exposure to ALF fragments may serve to enhance oxidative killing while limiting extracellular inflammatory responses.

Exposure of neutrophils to ALF fragments did not alter how ALF-*M.tb* is being recognized by neutrophils; this is in contrast to our previous studies where ALF driven modifications on the *M.tb* cell surface allowed this mycobacterium to be better recognized by neutrophils (3). When focusing on ALF fragment effects on the neutrophil’s capacity to handle intracellular growth alone, we found that, in this case, neutrophils partially lost their ability to control ALF-*M.tb* growth better. This could be a direct consequence of the twofold decrease in P–L fusion events observed in the presence of ALF fragments. While there is less phagosome maturation in the presence of ALF fragments, there is still intracellular control of ALF-*M.tb* by neutrophils, which could be partially explained by the increase of total ROS production induced by the presence of ALF fragments. This production is directly dependent on the presence of ALF fragments, as we did not observe ROS production during ALF-*M.tb* infection alone (3). Interestingly, in the presence of ALF fragments we found that neutrophils completely lost their ability to control the total (intracellular and extracellular) ALF-*M.tb* growth. This suggests a more unique and important role for intraphagosomal ROS in ALF-*M.tb* growth control.

In addition to intraphagosomal ROS, fusion of granules with the *M.tb*-phagosome may explain our observations of intracellular ALF-*M.tb* growth control. These intracellular granules are known to contain many types of antimicrobial effectors such as defensins, cathepsins, lactoferrin, and lysozyme (17). In this regard, ALF-modified *M.tb* and/or the released cell wall fragments could alter antimicrobial peptide production and delivery to the phagosomal compartment. While we showed that ALF-*M.tb* and ALF fragments enhance phagosome maturation of infected neutrophils, the approximate twofold decrease in phagosome maturation in the presence of ALF fragments suggests that other mechanisms (such as respiratory burst) may explain intracellular *M.tb* growth control.

Alveolar lining fluid fragments, alone or during *M.tb* infection, induced TNF expression and/or production by neutrophils. TNF has been shown to increase neutrophil activation and respiratory burst (27), as well as play a critical role in host control of *M.tb* growth (32). Interestingly, we found that when neutrophils were preactivated with TNF there was a delay in the neutrophil’s ability to control ALF-*M.tb* growth.

The alveolar space is an understudied environment in *M.tb* pathogenesis; however, it is crucial to define the first *M.tb*-host cells interaction that will determine the progression or control of the infection. Although neutrophils are a minor population of cells in the healthy lung, their role in driving innate immune responses is shown to be important in both primary infection and active TB disease. Our published studies indicate that exposure of *M.tb* to ALF allows neutrophils to better control the infection by increasing their intracellular killing mechanisms and reducing the extracellular killing mechanisms that could contribute to tissue damage. Our current studies support the concept that the release of antigenic *M.tb* cell wall fragments during the contact of *M.tb* with the lung mucosa could contribute to the initial stages and progression of the infection. The solubility and abundance of these ALF fragments defines several scenarios where host cells, including neutrophils, could find them and be differently primed to handle the infection in the alveolar space. First, neutrophils could be exposed to fragments alone where, under these conditions, neutrophils respond by increasing their total oxidative response, but do not secrete granule-mediators such as MPO and elastase, which could drive NET formation, surface receptor expression and exacerbate the oxidative burst. Second, in the alveolar space, neutrophils could only encounter ALF-exposed *M.tb*, and under these conditions, neutrophils could control infection minimizing tissue damage (3). Finally, another possibility is that neutrophils could encounter both ALF fragments and ALF-*M.tb*, which our data indicate could define a perfect symbiosis. It is in this scenario where the neutrophil could benefit from being able to recognize *M.tb* better (higher association), better control the infection by increasing phagosome maturation and the oxidative burst, while at the same time avoiding degranulation and NETosis, and minimizing tissue damage. In this context, infection in the presence of fragments reduces the production of IL-8 and MPO, inflammatory immune mediators, acting to further decrease an unwanted and potentially harmful influx and activation of neutrophils at the infection site. Conversely, *M.tb* could benefit, because as an intracellular pathogen *M.tb* gets recognized more rapidly and quickly taken up by resident neutrophils, allowing *M.tb* to bypass any neutrophil extracellular killing mechanisms. ALF fragments also drive TNF production, which may delay neutrophil activation and *M.tb* killing. In this context, our data also indicate that stimulation of neutrophils with ALF fragments boosts ROS while limiting degranulation, IL-8 release and restricting propensity of neutrophils to kill ALF-*M.tb* (upon TNF activation and total). Altogether such processes may promote silent *M.tb* replication in neutrophils rather than controlling *M.tb* growth in these myeloid cells.

Finally, when analyzing if exposure to ALF fragments alters crosstalk between neutrophils and macrophages, we found that secreted factors from primed ALF fragment-exposed neutrophils downregulated the activation status of macrophages, altering their surface expression of important phagocytic receptors such as the MR, and co-stimulatory molecules such as CD80/CD86. ALF fragment exposed neutrophils decreased MR expression on macrophages, which may limit both uptake and survival of *M.tb* within macrophages (20). In contrast, ALF fragment-exposed neutrophils had less effect on downregulation of major histocompatibility complex (MHC) class II (HLA-DR) on the surface of MDMs. Also these secreted factors from ALF fragment-exposed neutrophils did not induce macrophage secretion of important immune mediators such as TNF, IL-6, and IL-10. These data suggest that immunomodulators produced by neutrophils exposed to ALF fragments may influence the activation status of nearby resident macrophages (20). These findings are in contrast to LPS-primed neutrophils, which are shown to upregulate the MHC class II, and CD80 and CD86 co-stimulatory molecules on the macrophage surface, either by their secreted soluble factors such as TNF or by direct cell-to-cell contact [reviewed in Ref. (33)]. This observed immune response could be beneficial for *M.tb* whereby ALF-modified *M.tb* survives within neutrophils and is shielded from macrophage detection.

Our lab has previously shown that the lung mucosa enhances both macrophage and neutrophil-*M.tb* killing during infection (3, 4). Interestingly, we recently demonstrated that ALF-*M.tb* cell wall fragments further enhance human macrophage control of *M.tb* growth (14). This finding is in direct contrast to our observations in this current study, where ALF fragments fail to enhance neutrophil killing of *M.tb*. These differential observations may play a critical role during early *M.tb* infection whereby the first encounter between *M.tb* and a phagocyte in the alveolar space shapes the outcome of infection. Furthermore, ALF-*M.tb* fragments may promote *M.tb* survival within infected neutrophils and dually promote an encounter with de-activated macrophages if, and when, *M.tb* is released into the alveolar space.

Conversely, these observations of decreased macrophage activation in the presence of ALF fragment-exposed neutrophils may support a role for the lung mucosa in dampening inflammatory responses in the alveolar space. In this regard, ALF-*M.tb*-infected neutrophils in the presence of ALF fragments could control *M.tb* growth without the need for unnecessary local phagocyte activation.

Our results suggest the lung mucosa has the potential to impact establishment and early stages of *M.tb* infection; however, it is important to note that *M.tb* may encounter ALF at different stages of infection, such as following release from a dying host cell, as well as during active TB. In this type of scenario, it is likely the lung environment *M.tb* encounters may be altered due to changes in cellular composition and/or ALF. In support of this notion, we have published that aging (a TB-comorbidity) alters the composition of ALF components (34).

Overall, our results show that ALF-*M.tb* fragments could regulate the neutrophil innate response and contribute to the intracellular control of *M.tb* growth. These results indicate that ALF-induced *M.tb* fragments could stimulate *M.tb* local killing by resident neutrophils, by controlling the influx of neutrophils from the blood stream to the infection site as well as by limiting the immune response of resident phagocytes.

## Author Contributions

JS, JA, HK, LD, SS, EY, MW, S-HW, J-MB-L, and JT had substantial contributions to the conception or design of the work; or the acquisition, analysis, and/or interpretation of data for the studies. JS and JT drafted the work and/or revised it critically for important intellectual content.

## Conflict of Interest Statement

The authors declare that the research was conducted in the absence of any commercial or financial relationships that could be construed as a potential conflict of interest.
