# Supplementary material for: Mycobacterium tuberculosis Cell Wall Fragments Released upon Bacterial Contact with the Human Lung Mucosa Alter the Neutrophil Response to Infection
Source: Front Immunol. 2017 Mar 20;8:307. doi: 10.3389/fimmu.2017.00307 (PMC5357626; doi:10.3389/fimmu.2017.00307)

SUPPLEMENTAL MATERIAL

Supplemental Figure S1. ALF- or 0.9%NaCl-exposed *M.tb* cell wall fragments effect on human neutrophils. Neutrophil cell death following exposure to ALF- or 0.9%NaCl-*M.tb* fragments **(A&B)** and cell death of exposed-*M.tb* infected neutrophils in the presence of their respective fragments **(C&D)**. **(A)** Neutrophils ($2.5x10^5$) were exposed to 0.9%NaCl- or ALF-fragments at a range of MOEs (5:1, 10:1, 20:1 and 40:1) for 2h and 4h. Immediately following exposure, neutrophils were washed and stained for Annexin V (apoptosis) and 7-Aminoactinomycin D (live/dead). Data are n=2 with 2 ALFs. **(B)** Neutrophils ($2.5x10^5$) were exposed to 0.9%NaCl- or ALF-fragments at a MOE of 20:1 for 18h and the release of LDH was measured in cell supernatants. Percent cytotoxicity data are shown *vs* lysis positive control, data are n=1 with 3 ALFs. **(A&B)** NF: 0.9%NaCl-*M.tb* Fragments; NC: 0.9%NaCl-control; AF: ALF-*M.tb* Fragments; AC: Human ALF-control; R: Resting neutrophils; PC: Phorbol myristate acetate (PMA) 10 ng/mL-stimulated neutrophils. **(C)** Neutrophils ($1x10^6$) were infected with exposed-*M.tb* at an MOI of 1:1 for 30 min in the presence of their respective fragments. Immediately following infection (0.5h) and at time points of 1h, 3h, and 6h post-infection, neutrophils were washed and stained for Annexin V (apoptosis) and 7-Aminoactinomycin D (live/dead). Overall data from n=3 with 3 ALFs, where R: Resting (uninfected) neutrophils; NF: 0.9%NaCl-*M.tb* + 0.9%NaCl-Frag; NC: 0.9%NaCl-*M.tb* + 0.9% NaCl-Control; AF: ALF-*M.tb* + ALF-Frag; AC: ALF-*M.tb* + ALF-control. **(D)** Neutrophil monolayers were infected as in (C) and the release of LDH in cell supernatants indicative of cytotoxicity was measured every 30 minutes for up to 6 hours post-*M.tb* infection. Percent cytotoxicity data are shown *vs* lysis positive control, data are n=3 with 3 ALFs.

Supplemental Fig. S1

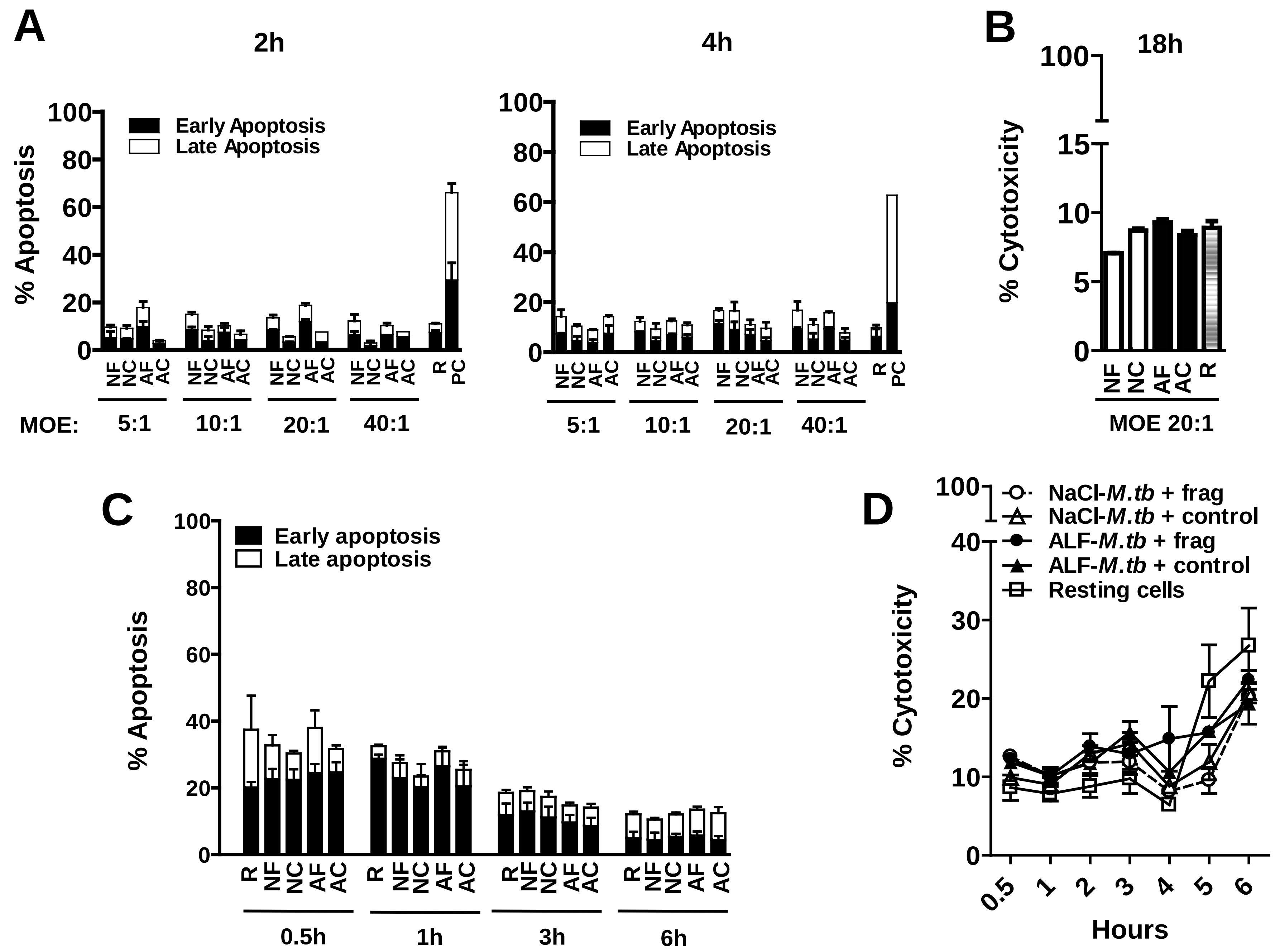

Supplement: Supplementary file 1 [file Presentation_1.PDF]
